# Orphan response regulator NnaR is critical for nitrate and nitrite assimilation in *Mycobacterium abscessus*

**DOI:** 10.3389/fcimb.2024.1411333

**Published:** 2024-05-24

**Authors:** Breven S. Simcox, Kyle H. Rohde

**Affiliations:** Division of Immunity and Pathogenesis, Burnett School of Biomedical Sciences, College of Medicine, University of Central Florida, Orlando, FL, United States

**Keywords:** *Mycobacterium abscessus*, nitrogen metabolism, nitrate, nitrite, orphan response regulator, NnaR

## Abstract

*Mycobacterium abscessus* (*Mab*) is an opportunistic pathogen afflicting individuals with underlying lung disease such as Cystic Fibrosis (CF) or immunodeficiencies. Current treatment strategies for *Mab* infections are limited by its inherent antibiotic resistance and limited drug access to *Mab* in its *in vivo* niches resulting in poor cure rates of 30-50%. *Mab’s* ability to survive within macrophages, granulomas and the mucus laden airways of the CF lung requires adaptation via transcriptional remodeling to counteract stresses like hypoxia, increased levels of nitrate, nitrite, and reactive nitrogen intermediates. *Mycobacterium tuberculosis* (*Mtb*) is known to coordinate hypoxic adaptation via induction of respiratory nitrate assimilation through the nitrate reductase *narGHJI*. *Mab*, on the other hand, does not encode a respiratory nitrate reductase. In addition, our recent study of the transcriptional responses of *Mab* to hypoxia revealed marked down-regulation of a locus containing putative nitrate assimilation genes, including the orphan response regulator *nnaR* (nitrate/nitrite assimilation regulator). These putative nitrate assimilation genes, *narK3* (nitrate/nitrite transporter), *nirBD* (nitrite reductase), *nnaR*, and *sirB* (ferrochelatase) are arranged contiguously while *nasN* (assimilatory nitrate reductase identified in this work) is encoded in a different locus. Absence of a respiratory nitrate reductase in *Mab* and down-regulation of nitrogen metabolism genes in hypoxia suggest interplay between hypoxia adaptation and nitrate assimilation are distinct from what was previously documented in *Mtb*. The mechanisms used by *Mab* to fine-tune the transcriptional regulation of nitrogen metabolism in the context of stresses e.g. hypoxia, particularly the role of NnaR, remain poorly understood. To evaluate the role of NnaR in nitrate metabolism we constructed a *Mab nnaR* knockout strain (*Mab_ΔnnaR_*) and complement (*Mab_ΔnnaR+C_*) to investigate transcriptional regulation and phenotypes. qRT-PCR revealed NnaR is necessary for regulating nitrate and nitrite reductases along with a putative nitrate transporter. Loss of NnaR compromised the ability of *Mab* to assimilate nitrate or nitrite as sole nitrogen sources highlighting its necessity. This work provides the first insights into the role of *Mab* NnaR setting a foundation for future work investigating NnaR’s contribution to pathogenesis.

## Introduction

The nontuberculous mycobacteria *Mycobacterium abscessus* (*Mab*) is an opportunistic pathogen afflicting the immunocompromised and individuals with pre-existing lung disorders such as cystic fibrosis (CF) (Brown-Elliott and Wallace, 2002; Olivier et al., 2003; Lee et al., 2015). CF patients are particularly vulnerable to bacterial infections due to reduced clearance of pathogens caused by viscous mucus buildup in the airways of the lungs and impaired innate immune responses leading to high rates of infections and morbidity (Lyczak et al., 2002; Chmiel and Davis, 2003). *Mab* is the most prevalent rapidly growing mycobacteria recovered from the lungs of CF patients, often causing deleterious effects such as lung function decline, resulting in extended hospitalization and in some cases exclusion from lung transplants (Olivier et al., 2003; Esther et al., 2010; Lopeman et al., 2019). Treatment options for *Mab* infections are limited due to inherent antibiotic resistance, resulting in cure rates of less than 50% (Hurst-Hess et al., 2017; Molina-Torres et al., 2018; Story-Roller et al., 2018; Lopeman et al., 2019). Like *M. tuberculosis* (*Mtb*), *Mab’s* ability to reside within macrophages and granulomas physically limits exposure to antibiotics and also promotes drug tolerance by driving metabolic and transcriptional remodeling, further complicating the treatment of these infections (Bernut et al., 2016; Peddireddy et al., 2017; Molina-Torres et al., 2018). *Mab* is also able to survive within the mucus that accumulates in the CF airway. In addition to creating a hypoxic microenvironment with a pO2 of ~1%, this mucus layer is characterized by increased levels of nitrite, nitrate and nitric oxide (Hassett et al., 2002; Worlitzsch et al., 2002; Robinson et al., 2017). Adaptation to these conditions along with other host-derived stressors requires extensive alterations in gene expression to allow *Mab* to establish persistent pulmonary infections. In *Mtb*, it is apparent that hypoxia survival strategies are inextricably linked to nitrogen metabolism via a shift to nitrate respiration as a way for bacilli to cope with low-oxygen microenvironments (Wayne and Hayes, 1998; Sohaskey and Wayne, 2003; Sohaskey, 2008). Our current understanding of how *Mab* adapts to these stresses *in vitro* or *in vivo* remains rudimentary at best. Several recent reports, including one from our group, highlighted the role of the *Mab* DosRS two-component system in the induction of a species-specific regulon in response to hypoxia (Belardinelli et al., 2022; Simcox et al., 2023). On the other hand, research examining the regulation of nitrogen metabolism in *Mab* is lacking. Prompted by our recent analysis of the hypoxia-induced transcriptional responses in *Mab* (Simcox et al., 2023), the current study provides additional evidence that *Mab* relies on strategies of nitrogen metabolism distinct from *Mtb* in response to *in vivo* relevant stresses.

The mechanisms for assimilation of nitrogen from nitrate and nitrite, which are readily accessible to mycobacteria during infection (Gouzy et al., 2014), have been well characterized in *Mtb* and *M. smegmatis* (*Msm*) however little is known about this facet of *Mab* host-pathogen interactions. Nitrate assimilation not only provides inorganic sources of nitrogen for the production of biomolecules but can also protect microbes against host iNOS (inducible nitric oxide synthase) generated nitric oxide (NO) an important factor used by the host to control infection (Kelm, 1999; Lundberg et al., 2004; Cunningham-Bussel et al., 2013; Gouzy et al., 2014). Host derived NO, after conversion to nitrate through auto-oxidation, can be exploited by *Mycobacterium* via the nitrate assimilation pathway resulting in the production of ammonium which can be stored as glutamate (Gouzy et al., 2014). *Mtb* constitutively expresses NarGHJI which serves as both an assimilatory nitrate reductase (NR), which converts nitrate to nitrite, and a respiratory NR allowing utilization of nitrate as a terminal electron acceptor (Sohaskey, 2008; Sohaskey and Modesti, 2009; Khan and Sarkar, 2012). The transcriptional and post-translational activation of NarK2, a putative H^+^/nitrate symporter (Giffin et al., 2012), under conditions that induced DosR (hypoxia, NO, CO), serves to induce nitrogen assimilation and respiration under hypoxic conditions (Voskuil et al., 2003; Khan and Sarkar, 2012). The excess, potentially toxic nitrite generated by NarGHJI is either extruded or further reduced to ammonium by the NirBD nitrite reductase (NiR) en route to storage or assimilation (Sohaskey and Wayne, 2003; Malm et al., 2009; Khan and Sarkar, 2012; Gouzy et al., 2014). *Msm* also encodes a constitutively expressed NarGHJI respiratory NR, however, unlike *Mtb*, it was shown to be non-functional for assimilation of nitrogen from nitrate (Sohaskey and Wayne, 2003; Malm et al., 2009; Cardoso et al., 2021). Instead, *Msm* relies on an unusual NADPH-dependent, diflavin-containing NR called NasN which is essential for nitrate utilization in aerobic conditions (Tan et al., 2020; Cardoso et al., 2021) as well as a NirBD ortholog to catalyze reduction of nitrite (Akhtar et al., 2013; Martin and Liras, 2019). Each of these components – *narGHJI, nirBD*, and *nasN* – along with a putative NarK nitrate/nitrite antiporter (MSMEG_0433) represent distinct operons or transcripts (Jenkins et al., 2013; Antczak et al., 2018). In contrast to *Mtb* and *Msm, Mab* does not encode a respiratory NarGHJI suggesting an inability to utilize nitrate as an alternative electron acceptor in low oxygen conditions. However, the *Mab* genome does include orthologs of other key components of nitrate/nitrite metabolism, including *narK3* (*Mab_3523c*), *nirBD* (*Mab_3522c-3521c*), *nnaR* (transcriptional regulator *Mab_3520c*) and *sirB* (siroheme ferrochelatase *Mab_3519c*). These five genes are arranged as a predicted operon that we found to be dramatically repressed in response to hypoxia in a DosR-independent manner (Simcox et al., 2023). Missing from this locus was a candidate NasN-type assimilatory NR. As detailed herein, we have identified a putative NasN ortholog (*Mab_2438)* that was co-regulated with the *narK3-sirB* operon (*Mab_3523c-3519c)* we predict catalyzes the reduction of nitrate to nitrite.

Both *Mycobacteria* and related *Streptomyces* rely upon an intricate network of regulators including two-component systems (TCS), serine-threonine protein kinases (STPKs), and orphan response regulators (ORR) to coordinate the integration of multiple cues to control nitrogen metabolism. GlnR, an ORR that can be activated by phosphorylation of Ser/Thr residues (Hong et al., 2007; Lin et al., 2014), is known as the master regulator of nitrogen metabolism in actinobacteria such as *Mycobacteria* and *Streptomyces* (Amin et al., 2012; Jenkins et al., 2013; He et al., 2023). *Mtb* GlnR is required for growth on nitrate and nitrite when these are the only nitrogen sources available and was shown to be a positive regulator of *nirBD* (Malm et al., 2009). Additionally, GlnR has been shown to crosstalk with the response regulators PhoP and MtrA which act as repressors of nitrogen metabolism through competition with GlnR for binding sites under nitrogen replete conditions (Rodriguez-Garcia et al., 2009; Sola-Landa et al., 2013; Martin and Liras, 2019; Zhu et al., 2019). As discussed in more detail below, an additional ORR, NnaR, with an unusual domain structure comprised of a HemD (uroporphyrinogen-III synthase) domain fused to a DNA-binding domain, is also implicated in nitrate and nitrite assimilation (Amin et al., 2012). Despite extensive studies examining *Mtb* and *Msm* transcriptional regulation of nitrogen metabolism and nitrate/nitrite assimilation, this remains a significant knowledge gap for *Mab* (Malm et al., 2009; Huang et al., 2015; Williams et al., 2015; Fan et al., 2024). Recently, the GlnR ortholog in *Mab* was shown to regulate *narK3*, *nirBD*, *glnA* (glutamine synthetase), and *amt* (ammonium transporter) although a role in the regulation of *Mab nasN* was not reported (Fan et al., 2024). The inactivation of GlnR in *Mab* led to an inability to form biofilms resulting in drug susceptibility dependent upon glutamine and glutamate availability (Fan et al., 2024). The role of other transcription factors in the regulation of nitrogen metabolism in *Mab* remains to be investigated. Notably, there have been no reports of the identification of a NnaR ortholog or its role in the use of nitrate/nitrite as nitrogen sources or detoxification of nitrite.

NnaR was first identified in *Streptomyces coelicolor* (*S. coelicolor*) as a GlnR target and co-activator with GlnR of nitrite reductase (*nirB*), nitrate transporter (*narK*) and nitrate reductase (*nasA*) (Amin et al., 2012). In the absence of NnaR, *S. coelicolor* displayed diminished capacity to reduce nitrite to ammonium resulting in a growth defect when nitrate or nitrite were used as sole nitrogen sources. However, nitrate was reduced to nitrite suggesting NnaR_Sc_ is not a regulator of nitrate reductase (Amin et al., 2012). Similar to *S. coelicolor*, *Msm* NnaR regulates key nitrogen assimilation genes and is required for use of nitrate and nitrite as sole nitrogen sources (Antczak et al., 2018). Subsequent research by Tan et al., determined GlnR_*Msm*_ is responsible for regulation of the assimilatory nitrate reductase NasN, confirming regulation of nitrate assimilation in *Msm* involves both GlnR (nitrate>nitrite) and NnaR (nitrite > ammonium) (Tan et al., 2020). The distinct roles for these two transcription factors in regulating different steps of nitrate assimilation were affirmed by EMSA data showing binding of NnaR to the *nirBD* promoter and GlnR binding upstream of *nasN* (Antczak et al., 2018; Tan et al., 2020). An ortholog of NnaR is also encoded by *Mtb* (Rv0260c), but little is known about it beyond its induction by multiple stress conditions including hypoxia, nutrient starvation, acid pH, and stationary phase (Vilcheze et al., 2022). The regulon and physiological role of *Mab* NnaR remains unknown, highlighting the need for further investigation of NnaR-mediated transcriptional regulation.

The goal of this study was to elucidate the previously unexplored role of *Mab* NnaR in gene regulation and nitrogen metabolism. We generated a *Mab nnaR* knockout strain (*Mab_ΔnnaR_*) and complement strain (*Mab_ΔnnaR+C_*) to further study NnaR and its function. Our work revealed that *Mab* NnaR regulates the *nark3-nirBD-nnaR-sirB* operon and *nasN* in a species-specific manner enabling nitrate and nitrite utilization. qRT-PCR analyses demonstrated that both loci were highly upregulated when nitrate or nitrite were the sole nitrogen sources, and with the exception of *sirB*, this gene induction was dependent on NnaR. The unexpected induction of *sirB* upon deletion of *nnaR*, which was restored to baseline levels in the complemented strain, suggests additional layers of regulation on this component of the operon. The inability of *Mab_ΔnnaR_* to regulate nitrogen assimilation genes resulted in growth defects when nitrate, and to a lesser degree nitrite, were used as the sole nitrogen source. This study highlights novel species-specific aspects of nitrogen metabolism in *Mab* and the critical role of NnaR and its downstream regulon in nitrate/nitrite utilization and detoxification. These findings support the hypothesis that NnaR is important for host adaptation and *Mab* pathogenesis.

## Methods

### Plasmid and *Mycobacterium abscessus* strain construction

*Mab*_*ΔnnaR*_ was engineered via recombineering as described by van Kessell and Hatfull in the strain *Mab* 390S obtained from the Thomas Byrd lab (Byrd and Lyons, 1999; van Kessel et al., 2008). In brief, an allelic exchange substrate (AES) was generated containing an apramycin resistance cassette flanked by ~600 nucleotides upstream and downstream of the *nnaR* gene (*MAB_3520c*) for homologous recombination. To construct the AES, the *nnaR* gene plus ~600 nucleotides upstream and downstream were PCR amplified and cloned into pCRBluntII-TOPO vector (Thermo Fisher) followed by round-the-horn PCR to remove the *nnaR* gene, retaining nucleotide overlap from genes upstream and downstream of *nnaR* (Li et al., 2011). After removal of *nnaR*, a phosphorylated apramycin resistance cassette was ligated between the upstream and downstream flanking regions. PCR amplification was then used to generate the AES consisting of the apramycin cassette flanked by upstream and downstream nucleotides. Mab::pJV53 competent cells induced with 0.02% acetamide for 4 hours were electroporated with 100 ng AES, recovered in 7H9 OADC media for 24 hours, and plated on 7H10 agar supplemented with apramycin 50 µg/ml. Complement strain, *Mab_ΔnnaR+C_* containing *nnaR* and *sirB* inserted downstream of the constitutive hsp60 promoter (P_hsp60_) in the episomal vector pVV16 was engineered using round the horn PCR of the vector (Li et al., 2011) and blunt ligation of the phosphorylated insert (Parikh et al., 2013). The *sirB* gene downstream of *nnaR* was included due to anticipated polar effects of inactivation of *nnaR.* This construct was introduced by electroporation into *Mab_ΔnnaR_*, followed by recovery in 7H9 OADC media for 24 hours and plating on 7H10 agar supplemented with kanamycin 50 µg/ml. *Mab nnaR* knockout was confirmed via PCR, sequencing and qRT-PCR. PCR screening entailed amplification of the apramycin resistance cassette or *nnaR* plus upstream and downstream flanking regions generating a product of 2,071 bp for *ΔnnaR* or a 2,287 bp product for WT *Mab* (refer to primer table for PCR screening, nnaR-F and nnaR-R). Sequencing of positive *nnaR* knockout clone was performed by Azenta.

Mycobacterial Protein Fragment Complementation (M-PFC) constructs were generated in the background plasmids pUAB100, pUAB200, and pUAB400 (Singh et al., 2006) using round the horn cloning and ligation. *Mycobacterium smegmatis* competent cells were electroporated with 100ng of each pUAB100:*dosS*/pUAB400:*dosR*, pUAB100:*dosS*/pUAB200:*nnaR* or pUAB100:*dosS*/pUAB400:*nnaR* at the same time and recovered in LB broth for 4 hours before plating on LB Agar supplemented with hygromycin B 50 µg/ml and kanamycin 50 µg/ml. Primers used for cloning are listed in **Supplementary Table S1**. Strains and plasmids are listed in **Supplementary Table S1**.

### Bacterial growth conditions and nitrogen supplementation

*Mab* cultures were grown in 7H9 OADC+.05% tyloxapol from glycerol stocks and incubated at 37° C while shaking. Growth kinetic studies under nitrogen limitation were carried out in Sauton’s nitrogen free minimal media (0.05% KH_2_PO_4_, 0.05% MgSO_4_, 0.2% citric acid, 0.005% ferric citrate, 0.2% glycerol, 0.0001% ZnSO_4_, 0.015% tyloxapol) supplemented with 2.5 mM sodium nitrate, 0.5 mM sodium nitrite or 2.5 mM ammonium sulfate as previously described (Jenkins et al., 2013; Antczak et al., 2018). Briefly, cultures were grown to mid-log phase in 7H9 OADC+0.05% tyloxapol washed twice with Sauton’s minimal media and then diluted to 0.2 OD followed by nitrogen supplementation. On days 0-5 cultures were serially diluted and spot plated on LB Agar for CFU enumeration.

### RNA extraction and qRT-PCR and operon validation

RNA was extracted as described by Rohde et al. (Rohde et al., 2007) in triplicate from cultures grown in 7H9 OADC+.05% tyloxapol or Sauton’s nitrogen free minimal media supplemented with nitrogen sources. Cultures were pelleted at 4,300 rpm for 10 minutes, resuspended in guanidine thiocyanate buffer, pelleted again at 12,000 rpm for 5 min, and stored at -80° C until processing. Thawed pellets were resuspended in 65° C Trizol then lysed using 0.1mM silicon beads in a BeadBeater at max speed for 1 minute 2x followed by cooling on ice for 1 minute between bead beating. Isolation of total RNA from Trizol lysates was performed using chloroform extraction and Qiagen RNeasy column purification. Total RNA was treated with Turbo DNase (Invitrogen) to eliminate DNA contamination. 50 ng/µl of total RNA was used to generate cDNA using iScriptTM cDNA synthesis kit (Bio-Rad) for qRT-PCR and RT-PCR. qRT-PCR reactions were carried out in a QuantStudio7 thermocycler using SYBR green as readout. Primers used for qRT-PCR are listed in **Supplementary Table S1**. To confirm whether genes in the putative *nark3-sirB* operon were transcribed as a single transcript, 400ng cDNA generated from RNA as described above was PCR amplified using primers that went across gene junctions (primers are listed in **Supplementary Table S1**) and analyzed by agarose gel electrophoresis.

### Mycobacterial protein fragment complementation asays

*Mycobacterium smegmatis* containing M-PFC constructs were grown to mid-log phase in LB 0.05% Tween 80 supplemented with hygromycin B 50 µg/ml and kanamycin 50 µg/ml were diluted to 0.0005 OD in same media. Cultures were then incubated in a 96-well plate for 48 hours with 2-fold serial dilutions of trimethoprim ranging from 200-3.125 µg/ml. After 48 hours 0.01% resazurin was added to wells and left to incubate for 4 hours. Data was collected using a Synergy 4 plate reader with fluorescence set to 530 nm excitation and 590 nm emission (Singh et al., 2006).

### Nitrite utilization

The ability for *Mab* to utilize nitrite was analyzed via colorimetric Griess reagent assay as described by Yang et., with minor modifications (Yang et al., 2016). On days 2 and 4, 50 µl of supernatant was added to a 96 well plate with 100 µl Griess Reagent (Thermo Fisher Scientific # 328670500) consisting of 1% sulphanilic acid in 5% phosphoric acid and 0.1% naphthylethylenediamine dihydrochloride. After plate was incubated at 37° C for 10 minutes absorbance was read at 535 nm on a Synergy 4 Plate reader. A standard curve with known nitrite concentrations was used to calculate concentrations of nitrite in supernatant.

## Results

### Identification and bioinformatic analysis of *Mab* orphan response regulator NnaR

**Figure 1:**
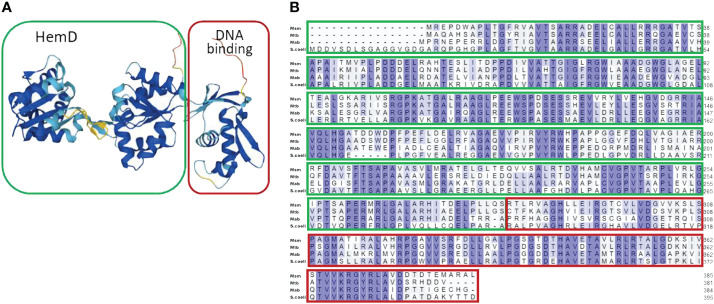
Bioinformatic analysis of NnaR domain structure and sequence alignment. Uniprot Bioanalysis Online Tool was used to generate the **(A)** Alpha-fold model of 3-D structure of *Mab* NnaR HemD-ORR protein. and **(B)** sequence alignment of *Mab* NnaR (MAB_3520c) compared to *Msm* NnaR (MSMEG_0432), *Mtb* (Rv0260c) and *S. coelicolor* NnaR (SCO2958). The N-terminal HemD domain is outlined in green, and C-terminal DNA-binding domain in red. Sequences for *Mab*, *Msm* and *Mtb* were obtained from the online data base Mycobrowser. *S. coelicolor* sequence was obtained from the online KEGG data base.

Our previous study investigating *Mab* transcriptional modulation during hypoxic adaptation revealed dramatic down-regulation of a gene locus containing an uncharacterized transcriptional regulator (*MAB_3520c*) that appeared to be an orphan response regulator (ORR) linked to multiple nitrogen assimilation genes (*MAB_3523c*, *MAB_3522c*, *MAB_3521c*) (Simcox et al., 2023). The down-regulation of these genes in response to hypoxia contrasts with reported transcriptional adaptations *Mtb* to hypoxia (Wayne and Hayes, 1998; Sohaskey and Wayne, 2003; Sohaskey, 2008) prompting our interest in the role of this ORR and nitrogen assimilation in *Mab*. Bioinformatic analysis of MAB_3520c using UniProt and Alphafold structural modeling revealed a C-terminal OmpR/PhoB-type winged helix DNA binding domain commonly found in response regulators of two-component systems (**Figure 1A**). However, lack of an adjacent histidine kinase gene and an unusual N-terminal domain containing a HemD domain rather than a receiver domain with aspartate phosphorylation sites indicated a unique ORR. This atypical ORR is orthologous to the NnaR response regulator of *S. coelicolor*, *Msm* and *Mtb* with 58%, 55%, and 54% sequence identity, respectively. Alignments between these organisms display homology between the C-terminal DNA binding domain of helix 3 and the unique HemD domain residues (**Figure 1B**). Due to homology within the DNA binding domain between organisms and published data documenting *S. coelicolor, Msm*, and *Mtb* NnaR binding motifs, we were able to justify using these orthologous motifs to identify *Mab* NnaR promoter binding sites (Amin et al., 2012; Antczak et al., 2018).

### The genomic and transcriptional organization of the NnaR regulon in *Mab*

**Figure 2:**
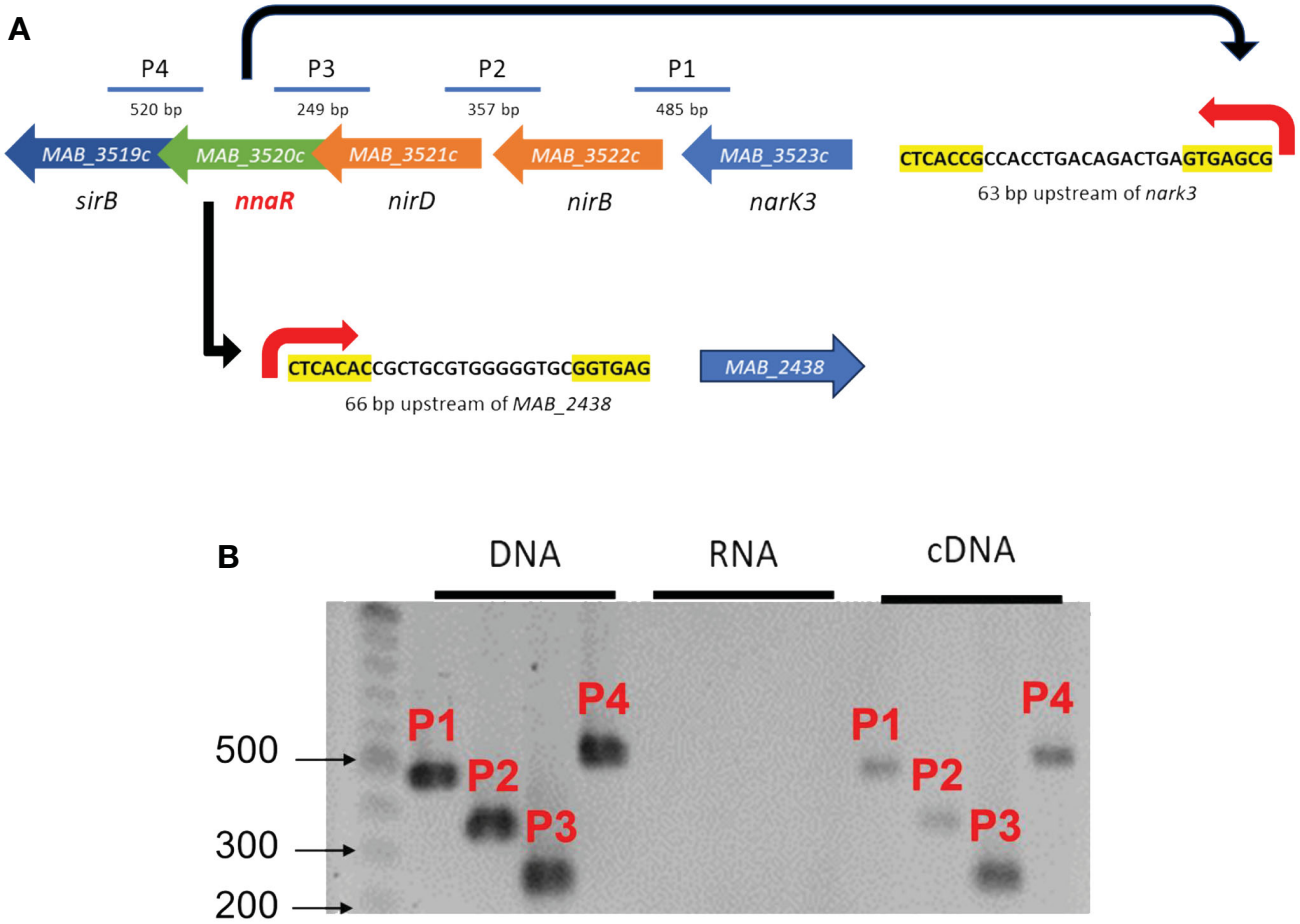
Genetic organization of the NnaR operon and regulon. **(A)** Schematic depicting nitrogen assimilation gene arrangement and NnaR binding sites discovered using DNA Pattern Find. Yellow highlighted nucleotides coincide with motifs used to find promoter region. P1-P4 indicates intergenic amplicons for RT-PCR. **(B)** Agarose gel of RT-PCR products P1-P4 amplified from DNA (positive control), RNA (negative control for DNA contamination), or cDNA.

Initially, we sought to leverage previously published NnaR binding sites to define a putative NnaR regulon in *Mab* via in silico analysis of the *Mab* ATCC_19977 genome using the DNA Pattern Find Tool (Amin et al., 2012). Using either the motif (CTCAC[A/C][Cg]._[13-16bp]_.GTGAG[CG][GA] based on predicted *S. coelicolor* and *Mtb* promoters or (CTCAC[A/C]._16bp_.[T/GGTGAG) reported by Antczak et al. for *Msm* (Antczak et al., 2018), only two predicted binding sites for *Mab* NnaR were identified. These NnaR promoters were positioned 63 bp and 66 bp upstream of the translational start sites of *narK3* and *MAB_2438*, respectively. *MAB_2438* encodes a previously uncharacterized protein annotated as a possible oxidoreductase (**Figure 2A**). Subsequent bioinformatic analysis of *MAB_2438* revealed 61% identity (71% similarity) with the recently described NADPH-dependent assimilatory NR *Msm* NasN encoded by *MSMEG_4206*, which had been erroneously annotated as a pseudogene due to an unconfirmed frame-shift mutation (Tan et al., 2020). Based on this observation, we will refer to the product of *MAB_2438* as NasN, which we noted was significantly shorter than its ortholog in *Msm* (MSMEG_4206 = 1351aa, *MAB_2438 = *1257aa). Primary amino acid sequence alignments of the two NasN orthologs (**Supplementary Figure S1**) revealed two reasons for this difference: 1) *MSMEG_4206* contains a ~75aa longer linker between the N-terminal nitrate reduction catalytic domain and C-terminal diflavin reductase domain (Tan et al., 2020), and 2) the annotated start codon for *MAB_2438* is likely incorrect. We predict that NasN*_Mab_* contains an additional 25 aa on the N-terminus based on homology with NasN_Msm_ and the presence of a critical [4Fe-4S] binding site. NnaR-mediated regulation of nitrate assimilation would be unique to *Mab* given the absence of NnaR binding sites upstream of *Msm nasN* as previously mentioned (Tan et al., 2020). We next sought to verify whether genes *nark3-nirBD-nnaR*-*sirB* are co-transcribed as an operon via RT-PCR using intergenic primers amplifying the 3’ end of one gene and the 5’ end of the next gene. Amplification of junctions between *nark3-nirB*, *nirB-nirD*, *nirD-nnaR* and *nnar-sirB* all yielded the expected bands in the DNA (positive control) and cDNA lanes, with no bands present in the RNA (no RT negative controls) lanes (**Figure 2B**). These data confirm that the five-gene *narK3*-*sirB* locus positioned downstream of a predicted NnaR binding site is expressed as a single mRNA transcript. This represents a species-specific organization of genes involved in nitrate/nitrite assimilation, compared to *Mtb* or *Msm* in which these genes are not arranged in a single operon (**Supplementary Figure S2**).

**Figure 3:**
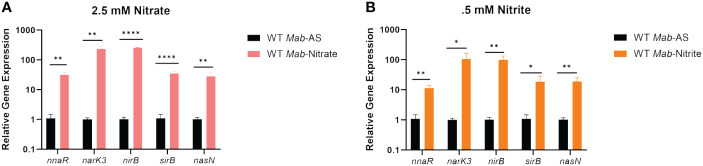
The NnaR regulon is induced when nitrate or nitrite are the sole sources of nitrogen. qRT-PCR was performed to confirm induction of *nnaR* regulon when nitrate **(A)** or nitrite **(B)** are the sole nitrogen sources. *Mab390S* supplemented with 2.5 mM ammonium sulfate (black bars), 2.5 mM nitrate (pink bars) and 0.5mM nitrite (orange bars). qRT-PCR data is representative of 3 experiments performed in duplicate. *P* values were calculated via t-test using GraphPad. *P-value <0.05, **P-value <0.01 ****P-value <0.0001.

We next investigated transcription of the *nark3-sirB* operon and *nasN* in the presence of nitrate or nitrite as sole nitrogen sources via qRT-PCR (**Figures 3A, B**). WT *Mab390S* cultures were supplemented with either 2.5 mM ammonium sulfate (AS) (control), 2.5 mM of sodium nitrate, or 0.5 mM of sodium nitrite in Sauton’s minimal media for 4 hours prior to RNA extraction. Sodium nitrite was used at a lower concentration due to toxicity at concentrations equivalent to AS and sodium nitrate (**Supplementary Figure S3**). qRT-PCR confirmed a 1-2 log induction of all genes within the *nark3-sirB* operon and *nasN* in the presence of nitrate (**Figure 3A**) and nitrite (**Figure 3B**) compared to the AS control, which bypasses the first two-steps of nitrate assimilation. These studies demonstrated the coordinated induction of the predicted NnaR regulon, including the *nark3-sirB* operon as well as *nasN*, specifically when nitrate or nitrite (compared to ammonium sulfate) are utilized as sole nitrogen sources.

### NnaR-dependent expression of the *narK3*-*sirB* operon and *nasN*

**Figure 4:**
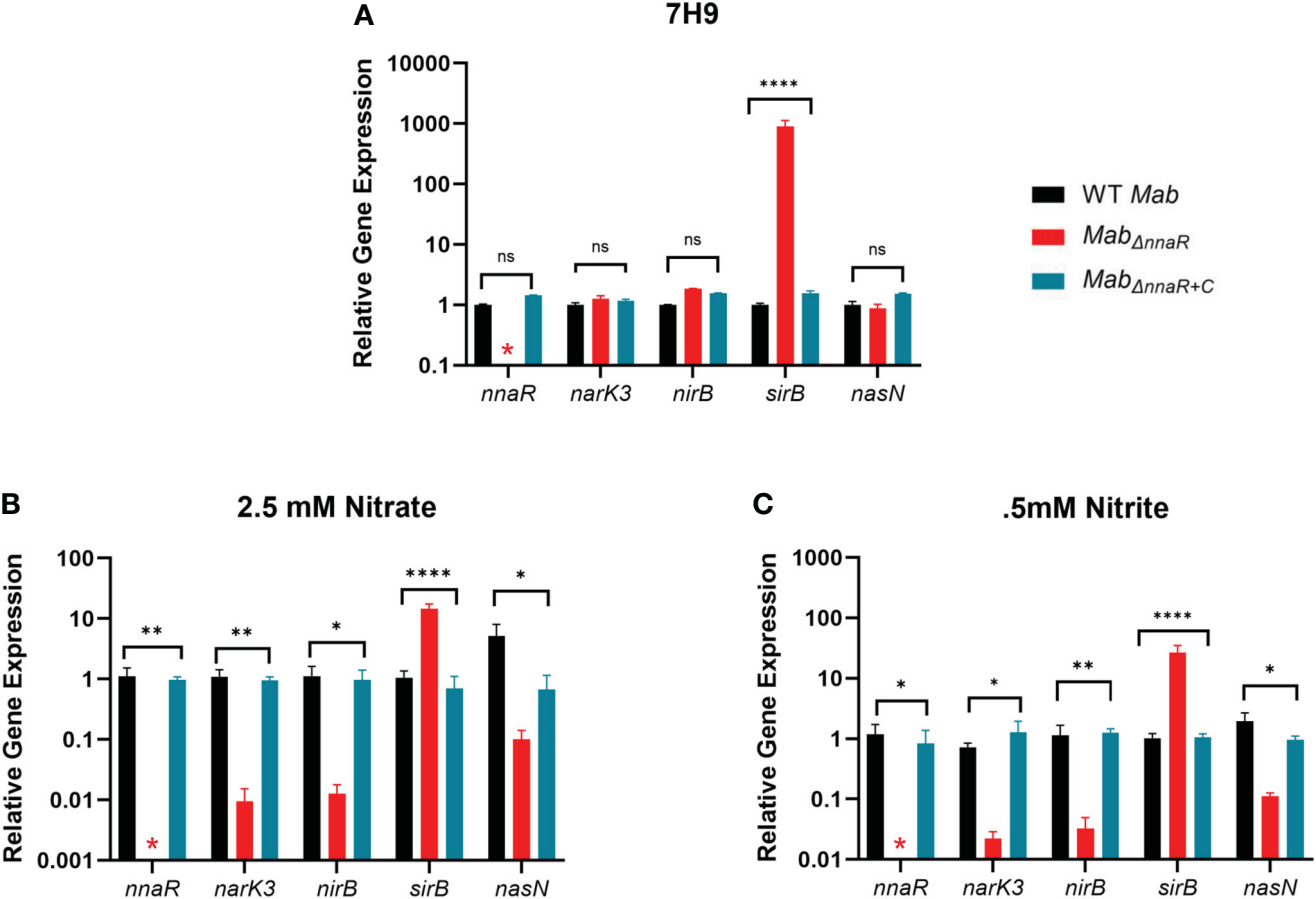
NnaR regulates the *nark3-sirB* operon and *nasN*. qRT-PCR was performed to confirm the deletion and restoration of *nnaR* in *Mab*_*ΔnnaR*_ and *Mab*_*ΔnnaR+C*_, respectively, and effect on predicted regulon in 7H9 media **(A)**, in minimal media supplemented with nitrate **(B)** or nitrite **(C)**. WT *Mab 390S* (black)*, Mab*_*ΔnnaR*_ (red) and *Mab*_*ΔnnaR+C*_ (blue). Data is representative of 3 experiments performed in duplicate. *P* values were calculated via one-way ANOVA using GraphPad. Red stars indicate C_t_ values were not detected for *Mab nnaR* in the mutant strain. *P-value <0.05, **P-value <0.01 ****P-value <0.0001. ns denotes samples that were not significantly different than WT.

To further investigate the role of NnaR, a *Mab nnaR* knockout strain (*Mab_ΔnnaR_*) was generated via recombineering (van Kessel and Hatfull, 2007). Anticipating polar effects, a corresponding complement strain (*Mab_ΔnnaR+C_*) containing both *nnaR* and *sirB* constitutively expressed from the *hsp60* promoter in the episomal vector pVV16 was constructed (van Kessel et al., 2008; Parikh et al., 2013). In addition to PCR (**Supplementary Figure S4**) and sequencing, qRT-PCR was used to confirm the knockout and complement via expression levels of *nnaR* and the effect on genes within the predicted *nnaR* regulon (**Figure 4A**). Data from log-phase cultures in 7H9 media confirmed *nnaR* transcripts were not detectable in *Mab_ΔnnaR_* with restoration to WT levels in *Mab_ΔnnaR+C_*. Consistent with the predicted role for NnaR as an activator of its own operon plus *nasN*, we expected loss of NnaR to lead to downregulation of its regulon. Surprisingly, gene expression levels for *narK3*, *nirBD and nasN* in all three strains grown in 7H9 media were unaltered in the absence of NnaR, despite the presence of a NnaR binding site upstream *nark3* and *nasN*. Closer analysis of qRT-PCR raw data showed high C_t_ values for all genes, indicating minimal expression of these genes under nitrogen replete conditions. This observation is consistent with *Msm* NnaR studies which found no difference in the expression levels of *narK* and *nirB* in the absence of NnaR when grown in nitrogen replete media (Antczak et al., 2018). In contrast to WT levels of *nark3*, *nirB* and *nasN*, *sirB* exhibited significant ~3-log up-regulation in the Δ*nnaR* mutant strain, which was restored to WT levels in the complement strain. Thus, our data indicate that the basal expression of *nnaR* and associated nitrate/nitrite assimilation genes is low in WT *Mab* under nitrogen replete conditions such that the absence of NnaR had little effect in gene expression. The unusual expression pattern of *sirB*, which appears to be cotranscribed with the *narK3-sirB* operon but suppressed by NnaR, points to the possibility of additional internal promoters and/or additional layers of regulation acting on *sirB*.

Since the absence of NnaR had no effect on gene expression levels in nitrogen rich media, we evaluated gene expression between *Mab390S* and *Mab_ΔnnaR_* under inducing conditions with nitrate or nitrite as the sole nitrogen sources. Briefly, cultures were incubated in minimal media supplemented with 2.5 mM AS, 2.5 mM sodium nitrate or 0.5 mM sodium nitrite for 4hr prior to RNA isolation and qRT-PCR analysis. During growth on defined inorganic nitrogen sources, the absence of NnaR resulted in a significant down-regulation (≥ 1 log) or lack of induction of *nark3*, *nirB* and *nasN*, with restoration to wild-type levels in *Mab_ΔnnaR+C_* (**Figures 4B, C**). Consistent with our observations in 7H9 media, *sirB* transcript levels in *Mab_ΔnnaR_* remained elevated in nitrate or nitrite supplemented cultures, with restoration to WT levels in *Mab_ΔnnaR+C_*. As noted above, this lack of correlation between the transcription profiles of *sirB* and upstream genes appears inconsistent with their co-transcription as part of an operon. Decreased transcription of *nark3*, *nirBD*, and *nasN* in the mutant strain under inducing conditions affirms that NnaR mediates upregulation of its regulon in response to nitrate and nitrite. Induction of a putative assimilatory nitrate reductase *nasN* represents a species-specific role for NnaR*_Mab_* since NnaR*_Msm_* does not appear to regulate *nasN* (Tan et al., 2020). This data provides the first insights regarding regulation of nitrogen assimilation for *Mab* outside of what has been previously published for GlnR (Fan et al., 2024).

### NnaR is required for survival on nitrate or nitrite

**Figure 5:**
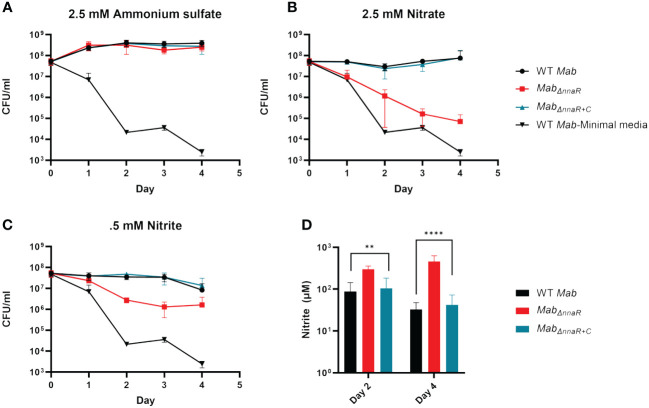
NnaR is responsible for nitrite utilization and growth when nitrate and nitrite are sole sources of nitrogen. Growth kinetics in minimal media supplemented with various nitrogen sources was assessed via serial dilutions, spot plating and enumeration of CFU/ml on days 0-4. Cultures supplemented with **(A)** ammonium sulfate, **(B)** nitrate or **(C)** nitrite. WT *Mab* (black circle), *Mab_ΔnnaR_* (red square), *Mab_ΔnnaR+C_* (blue triangle), WT *Mab-*Minimal Media (serving as negative control-no nitrogen supplementation, black upside down triangle). Griess assay **(D)** was performed to measure nitrite accumulation on day 2 and 4. *Mab 390S* (black bars)*, Mab_ΔnnaR_* (red bars) and *Mab_ΔnnaR+C_* (blue bars). Data reflects 3 independent experiments performed in triplicate. ** P-value <0.01, **** P-value <0.001.

Based on our data revealing NnaR-dependent upregulation of genes linked to nitrogen metabolism, we next investigated whether the absence of NnaR affects growth when nitrate or nitrite are the sole sources of nitrogen. Cultures of *Mab390S*, *Mab_ΔnnaR_*, *Mab_ΔnnaR+C_* were grown in minimal media or minimal media supplemented with 2.5 mM AS, 2.5 mM sodium nitrate or 0.5 mM sodium nitrite, with CFUs enumerated daily through day 4 (**Figure 5**). The slight initial increase in CFU of *Mab390S* cultures supplemented with AS which was sustained until day 4, in contrast to the ~4-log decline in CFU observed in minimal media alone (**Figure 5A**), affirms that *Mab* can utilize AS as a sole nitrogen source. Notably, the growth of *Mab_ΔnnaR_* in AS was indistinguishable from WT (**Figure 5A**) indicating that NnaR is not required when the first two-steps of nitrate assimilation (nitrate>nitrite>ammonium) are bypassed by providing ammonium as the sole nitrogen source (Cardoso et al., 2021). When supplemented with nitrate or nitrite, on the other hand, the WT and complement either maintained a steady number of CFU (nitrate) or decreased slightly (nitrite) (**Figures 5B, C**). We suspect the slight decline in CFU for nitrite supplemented cultures of strains expressing NnaR is due to nitrite toxicity resulting in growth inhibition, a phenomenon also observed in *Mtb* (Cunningham-Bussel et al., 2013). As predicted, the survival of *Mab_ΔnnaR_* was severely impaired when nitrate was the only nitrogen source, with a final CFU difference of ~3 logs on day 4 (**Figure 5B**). This verified the importance of NnaR in nitrate assimilation. The dramatic *Mab_ΔnnaR_* phenotype observed on day 4 with nitrate supplementation was less pronounced with nitrite supplementation (**Figure 5C**). However, a log difference was observed on day 2 and 3 when comparing *Mab_ΔnnaR_* to WT and complement with nitrite as the sole nitrogen source (**Figure 5C**). Due to decreased CFU from day 3 to day 4 in WT and complement in nitrite and a plateau in CFU of *Mab_ΔnnaR_* during the same period, there was only a difference of 0.5 log in CFU by the end of the assay (**Figure 5C**). Although the reason why loss of NnaR had a less deleterious effect on growth/survival of *Mab_ΔnnaR_* on nitrite versus nitrate is unclear, it is evident that NnaR plays a critical role in nitrogen assimilation and/or nitrite detoxication when nitrite is the sole nitrogen source available. To biochemically validate the role of NnaR in nitrite utilization, we compared the ability of WT, *Mab_ΔnnaR_*, and *Mab_ΔnnaR+C_* to use nitrite as a nitrogen source via Griess assay. Briefly, nitrite concentrations in supernatants taken from the growth kinetic study were measured on day 2 and day 4 to gauge nitrite utilization by *Mab* strains. Nitrite concentrations in the supernatant of *Mab_ΔnnaR_* were significantly higher than WT and complement at both day 2 and 4 (**Figure 5D**), affirming a reduction in the ability to metabolize nitrite in the absence of NnaR. Further studies are required to elucidate the biochemical functions and physiological roles of NnaR regulated enzymes in *Mab* nitrogen metabolism.

In summary, we have identified an orphan response regulator designated NnaR based on homology with recently characterized ortholog in *Msm*, its unusual domain structure (fusion of HemD domain with DNA binding domain), and critical role in Nitrate/Nitrite Assimilation Regulation (NnaR). It is encoded in a species-specific operon that is downregulated by hypoxia (Simcox et al., 2023) but highly induced by inorganic nitrogen sources, specifically nitrate and nitrite. Our data indicated that NnaR*_Mab_* activates transcription of a six gene regulon responsible for reduction of nitrate to nitrite (NasN) and nitrite to ammonia (NirBD), transport of nitrate and/or nitrite (NarK3), and synthesis of the siroheme cofactor for nitrite reductase. Finally, NnaR is required for the utilization of nitrate and nitrite as sole nitrogen sources and detoxification of nitrite, suggesting an important role for this transcription factor in *Mab* host adaptation and pathogenesis.

## Discussion

*Mab* persistence within the hostile niches of macrophage phagosomes, granulomas, and mucus-laden CF lung requires intricate gene regulation to combat low oxygen tension, reactive nitrogen intermediates (RNI), nutrient depletion, and exposure to excess nitrate and nitrite. Given the capacity of *Mtb* to adapt to similar niches and presence of many orthologous virulence factors and transcriptional regulators, it is tempting to extrapolate from available *Mtb* data to understand *Mab* gene regulation and function. However, emerging data from our lab and others highlights significant species-specific differences in transcriptional regulatory networks and strategies of adapting to *in vivo* conditions that urge caution when doing this. Unique strategies of *Mab* host adaptation and persistence stem from its distinct repertoire of genes compared to *Mtb* (Wee et al., 2017), variable genomic arrangements of the same genes, as well as shared orthologous transcription factors that control species-specific regulons (Gerasimova et al., 2011; Miranda-CasoLuengo et al., 2016; Bryant et al., 2021; Simcox et al., 2023).

In *Mtb*, the overlap and integration of genes involved in adaptation to hypoxia and nitrate metabolism, not only as a means of alternative respiration but also to defend against host generated RNI, are well-documented (Sohaskey, 2008; Malm et al., 2009; Akhtar et al., 2013; Gouzy et al., 2014; Vilcheze et al., 2022). On the other hand, relatively little is known about mechanisms used by *Mab* to coordinate responses to these two intertwined host cues. Our interest in nitrate assimilation in *Mab* and the interplay with hypoxia was sparked by our recent observation that genes implicated in nitrate assimilation are downregulated under hypoxic conditions (Simcox et al., 2023). This data contrasts with the response of *Mtb* to hypoxia, including upregulation of *narK2* and *nirBD* (Sohaskey, 2008; Malm et al., 2009; Akhtar et al., 2013; Gouzy et al., 2014), which facilitates utilization of nitrate as a terminal electron acceptor via reduction by the constitutively expressed *narGHIJ*, which acts as both a respiratory and assimilatory nitrate reductase (NR) (Sohaskey and Wayne, 2003; Malm et al., 2009; Gouzy et al., 2014). The resulting nitrite produced by NR could then be extruded by the putative nitrite transporter NarK2 to avoid toxicity or be further reduced to ammonium via the nitrite reductase *nirBD* under nitrogen-limited conditions (Malm et al., 2009; Gouzy et al., 2014). This strategy is not an option for *Mab*, due to the conspicuous lack of a NarGHJI-like respiratory NR. Our recently reported RNAseq analysis of *Mab* hypoxia adaption showed down-regulation of two response regulators implicated in nitrate assimilation, *MAB_0744* (*glnR*) and *MAB_3520c* (*nnaR*), in addition to the five additional genes of the NnaR regulon (this study), which may serve to reduce protein synthesis and aid transition to slowed growth under stressful conditions (Simcox et al., 2023).

In addition to species-specific regulation of the nitrate metabolism apparatus under hypoxic conditions, the genomic organization of genes predicted to be involved in this process is unique in *Mab.* The genes encoding proteins required for nitrate/nitrite transport (NarK3), reduction of nitrite to ammonia (NirBD), an ORR associated with nitrate assimilation (NnaR), and synthesis of the siroheme cofactor for NirBD (SirB) are clustered in the *MAB_3523c-MAB_3519c* locus, with the *nasN* NiR gene located elsewhere. The overlap between open reading frames of *nirB-nnaR* and *nnaR-sirB* suggest close translational coupling of these components (Huber et al., 2019). All five genes appear to be transcribed as a single unit, as evidenced by our RT-PCR analysis and tight co-regulation under hypoxia and nitrate/nitrite supported growth (**Figures 3A, B**). However, we cannot rule out the possibility of additional transcripts of subsets of genes from internal promoters. While *narK3, nnaR*, and *sirB* are arranged contiguously in *Mtb* and *Msm*, the *nirBD* operon is upstream and oriented divergently and nitrate reductases (*narGHJI-Mtb* and *nasN-Msm*) are located elsewhere (**Supplementary Figure S2**). Following identification of an uncharacterized orphan response regulator designated NnaR embedded within this operon, our focal point became elucidating its role in *Mab* gene regulation and nitrate assimilation.

In silico analyses identified only 2 putative NnaR binding sites in the *Mab* genome - upstream of *nark3-sirB* operon and *nasN* (**Figure 2A**) – suggesting a small regulon of genes with focused roles on nitrate and nitrite metabolism. This is comparable to the small repertoire of genes reportedly controlled by the *S. coelicolor* ortholog shown to regulate *narK, nirAB* (NiR), and *nasA* (assimilatory NR) (Amin et al., 2012). In *Msm*, NnaR controls a slightly larger geneset comprised of seven mRNA transcripts (four single genes, 3 operons), however only 3 target loci (*narK3, nirBD, nasN*) have corresponding orthologs in *Mab* (Antczak et al., 2018). The coordinated induction of the *nark3-sirB* operon and *nasN* during growth on inorganic nitrogen sources is consistent with regulation by the same transcription factor, NnaR. It is also worth noting that the dramatic ~20- to 200-fold induction of the NnaR regulon (**Figures 4B, C**) is in reference to equimolar concentrations of nitrogen in the form of ammonium sulfate. This would suggest that NnaR-mediated transcriptional activation is not in response to low nitrogen levels overall but is somehow specific to nitrate and nitrite. Based on these observations, the lack of induction of *nasN*, *nark3*, and *nirBD* in *Mab_ΔnnaR_* during growth on nitrate or nitrite was not surprising. However, the upregulation of *sirB* in the absence of *nnaR* in both nitrogen replete and deplete conditions was unexpected (**Figure 4**). This is inconsistent with *sirB* being expressed only by NnaR-activated co-transcription with the four upstream genes. An initial hypothesis we considered was upregulation due to read-through from the promoter of the apramycin^R^ cassette used to inactivate *nnaR*. However, restoration of *sirB* to wild-type levels when both *nnaR* and *sirB* were constitutively expressed in trans argues against this. If anything, an additional copy of *sirB* on the complement construct (included because we anticipated a polar negative effect) was expected to boost *sirB* expression in the complement, not restore levels to normal. It is possible that deletion of *nnaR* disrupts the proposed translational coupling with *sirB* (despite care taken not to delete the overlapped 5’ end of *sirB*) such that diminished SirB activity triggers compensatory upregulation of *sirB.* SirB is a ferrochelatase responsible for catalyzing the last step of siroheme synthesis, a cofactor required for the activity of nitrite reductase (NirBD) and sulfite reductases (Tripathy et al., 2010; Dailey et al., 2017; Pennington et al., 2020). This would require a second internal promoter able to drive expression of *sirB* alone (or with additional upstream genes since putative promoter cannot be within *nnaR)* activated by a transcription factor that is induced by NnaR. More research will be needed to understand this unexpected regulation of *sirB* and the role of NnaR.

The mechanism by which NnaR senses nitrate and nitrate, either directly or indirectly, and becomes activated to promote transcription of its regulon remains a mystery. No studies of the better characterized orthologs in *S.coelicolor*, *Msm*, or *Mtb* have addressed this question. We initially hypothesized that NnaR activation may be mediated by DosS, the sensor histidine kinase (HK) partner of the response regulator (RR) DosR, known to sense hypoxia, NO, and CO in *Mtb* (Voskuil et al., 2003, 2004; Shiloh et al., 2008; Sivaramakrishnan and de Montellano, 2013; Zheng et al., 2017). This was based on a report by Gautam et al. of potential protein-protein interactions (PPI) between DosS and the *Mtb* NnaR ortholog Rv0260 (Gautam et al., 2019). Using the same M-PFC (Mycobacterial Protein Fragment Complementation) method (Singh et al., 2006), we were able to detect *Mab* DosS-DosR PPI, but were unable to detect an interaction between *Mab* DosS and NnaR (**Supplementary Figure S5**). This is perhaps not surprising given the other species-specific differences we have noted between *Mtb* and *Mab* in their responses to hypoxia and nitrogen limitation. Further in silico analysis also failed to identify a receiver domain with aspartate residues likely to serve as phosphorylation sites (data not shown) suggesting NnaR is not likely to be activated by a non-cognate HK. Alternative hypotheses include NnaR activation by serine/threonine kinases as observed with the ORR GlnR (Hong et al., 2007; Lin et al., 2014; Amin et al., 2016), or self-activation via the unique HemD domain. The domain architecture of NnaR consisting of an N-terminal HemD domain (also known as a uroporphyrinogen III synthase) fused to a C-terminal OmpR DNA binding domain is restricted to Actinomycetes (Amin et al., 2012). Uroporphyrinogen III synthase is responsible for the cyclization of hydroxymethylbilane (HMB) which forms uroporphyrinogen III (UroPIII), a precursor for vitamin B12 and siroheme. It is worth noting that the HemD domain of NnaR in *S.coelicolor* is catalytically inactive (Schubert et al., 2008; Tripathy et al., 2010; Amin et al., 2012; Dailey et al., 2017). The role of the NnaR HemD domain remains unclear but it is plausible it acts as a sensing domain for HMB or UroPIII and self-activates resulting in conformational changes in the DNA-binding domain as evidenced by the self-modulating RRs, JadR1 and RedZ of streptomycetes (Gao et al., 2019).

This study provides clear evidence that *Mab* utilizes species-specific strategies of nitrogen assimilation when nitrate or nitrite are the only sources of nitrogen and employs the ORR NnaR in distinct ways compared to *Msm* and *Mtb*. This is illustrated by confirmation of a unique operon containing genes *narK3-nirBD-nnaR-sirB*, regulation of this operon and *nasN* by NnaR, and the importance of NnaR for survival when nitrate or nitrite are the sole sources of nitrogen. There are still unanswered questions regarding the regulation of nitrogen metabolism in *Mab*, and the role of NnaR in particular, that remain to be addressed. One key knowledge gap is the degree of regulatory overlap and interaction with GlnR, which typically controls larger regulons involved in nitrogen metabolism and many other processes. Although Fan et al. did report that *Mab* GlnR regulates 8 genes associated with nitrogen metabolism, they only queried 9 promoters by ChIP-qPCR (Fan et al., 2024). The full extent of the GlnR regulon in *Mab* remains to be determined. Other relevant questions include how hypoxia triggers down-regulation of the NnaR regulon, what is the function of the HemD domain, what underlies the unusual regulation of *sirB*, and what signaling mechanisms contribute to NnaR activation. These data revealing a species-specific role for *Mab* NnaR and its significance in nitrate assimilation establishes a premise for future studies exploring its role in persistence within host environments such macrophage phagosomes, granulomas, and mucus-laden airways affected by CF.

## Data availability statement

The original contributions presented in the study are included in the article/**Supplementary Material**, further inquiries can be directed to the corresponding author/s.

## Author contributions

BS: Conceptualization, Data curation, Formal analysis, Investigation, Methodology, Visualization, Writing – original draft, Writing – review & editing. KR: Conceptualization, Data curation, Formal analysis, Methodology, Project administration, Resources, Supervision, Visualization, Writing – original draft, Writing – review & editing.

## Supplementary material

Supplementary Figure 1 NasN sequence alignment. Uniprot sequence amino acid sequence alignment of NasN orthologs MSMEG_4206 and MAB_2438. Conserved amino acids are highlighted in blue. The annotated versus predicted translational start sites for MAB_2438 NasN are indicated by red arrows.

Supplementary Figure 2 Nitrate assimilation gene organization in *Mab*, *Mtb*, and *Msm.*

Supplementary Figure 3 Growth kinetics of Mab390S at different concentrations of inorganic nitrogen sources. Growth kinetics were measured by OD_600nm_ on day 4 of growth in minimal media supplemented with **(A)** 2.5 mM, 5 mM, and 10 mM ammonium sulfate **(B)** 0.5 mM, 1 mM, 2.5 mM sodium nitrite **(C)** 2.5 mM, 5 mM, and 10 mM sodium nitrate.

Supplementary Figure 4 PCR confirmation of nnaR mutant. PCR was used to screen for nnaR mutants via amplification of NnaR or Apramycin resistance cassette plus upstream and downstream flanking region. L1) DNA ladder; L2) WT Mab, 2287 bp amplicon; L3) negative clone; L4) ΔnnaR mutant, 2071 bp amplicon.

Supplementary Figure 5 MPFC assay displays lack of interaction between NnaR and DosS. Minimum inhibitory concentration of trimethoprim (TRIM) was analyzed to measure interaction of DHFR_1,2_ and DHFR_3_ indicative of PPI. Black circle) pUAB100-dosS-(DHFR_1,2_ on C-term):pUAB200-dosR-(DHFR_3_ on C-term) Red square) pUAB100-dosS-(DHFR_1,2_ on C-term):pUAB200-nnaR-(DHFR_3_ on C-term) Turquoise triangle) pUAB100-dosS-(DHFR_1,2_ on C-term):pUAB400-nnaR-(DHFR_3_ on N-term) Blue triangle) pUAB300-GCN4-(DHFR_1,2_ on C-term):pUAB400-GCN4-(DHFR_3_ on N-term) Gray Diamond) empty vector.
