# Supplementary figures and images for: Orphan response regulator NnaR is critical for nitrate and nitrite assimilation in Mycobacterium abscessus
Source: Front Cell Infect Microbiol. 2024 May 24;14:1411333. doi: 10.3389/fcimb.2024.1411333 (PMC11162112; doi:10.3389/fcimb.2024.1411333)

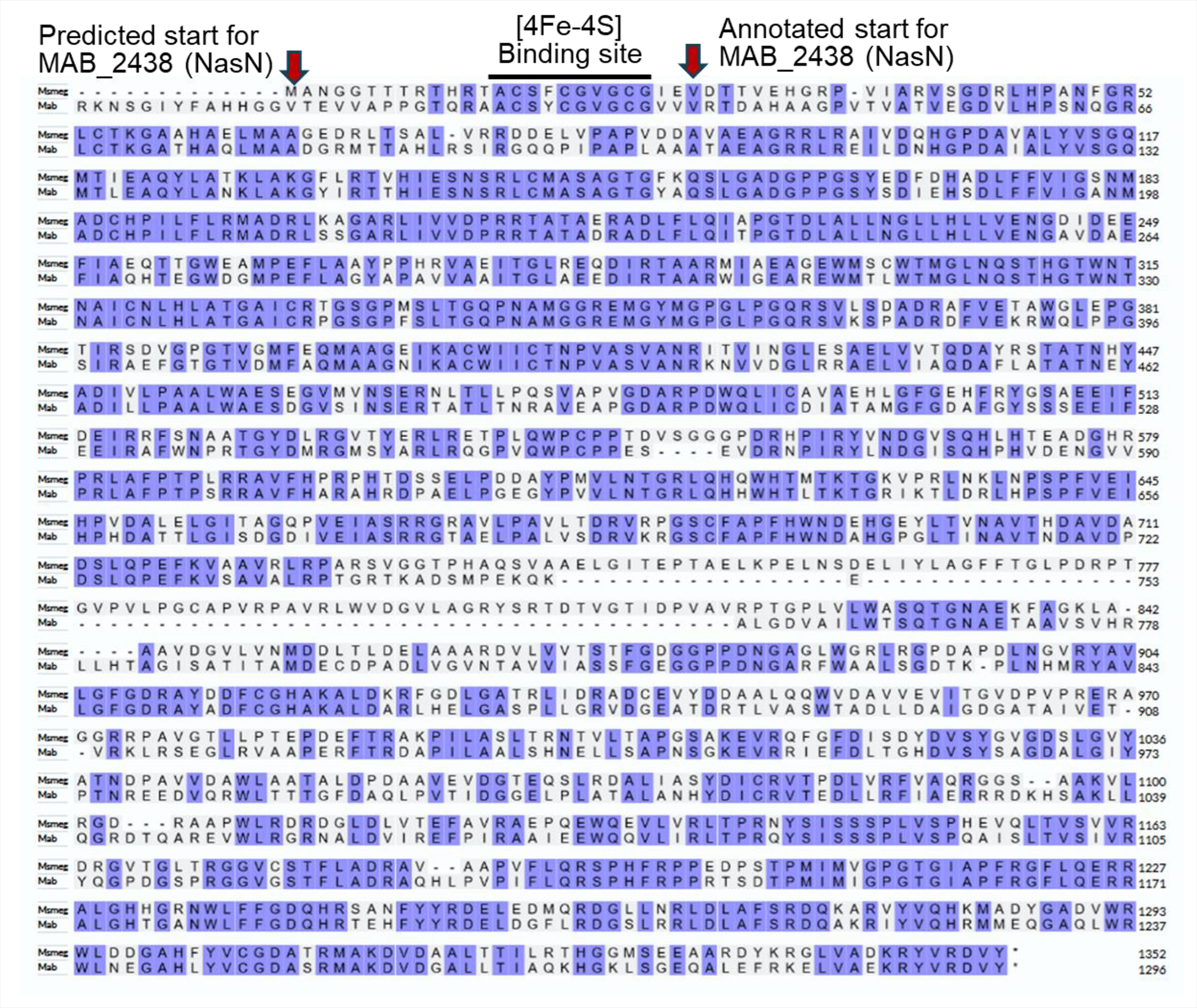

Supplement: Supplementary Figure 1 — NasN sequence alignment. Uniprot sequence amino acid sequence alignment of NasN orthologs MSMEG_4206 and MAB_2438. Conserved amino acids are highlighted in blue. The annotated versus predicted translational start sites for MAB_2438 NasN are indicated by red arrows. [file Image_1.tif]

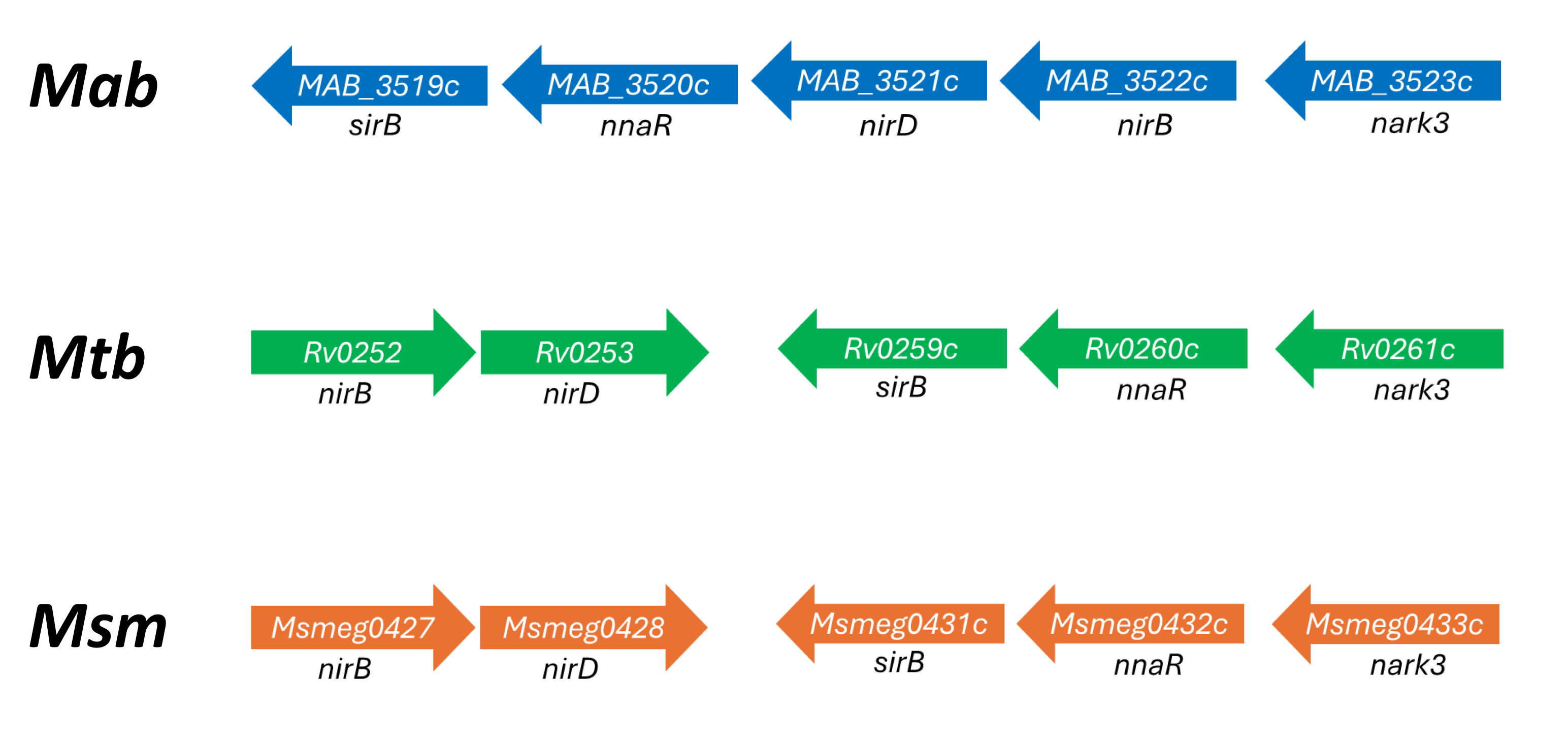

Supplement: Supplementary Figure 2 — Nitrate assimilation gene organization in Mab, Mtb, and Msm. [file Image_2.tif]

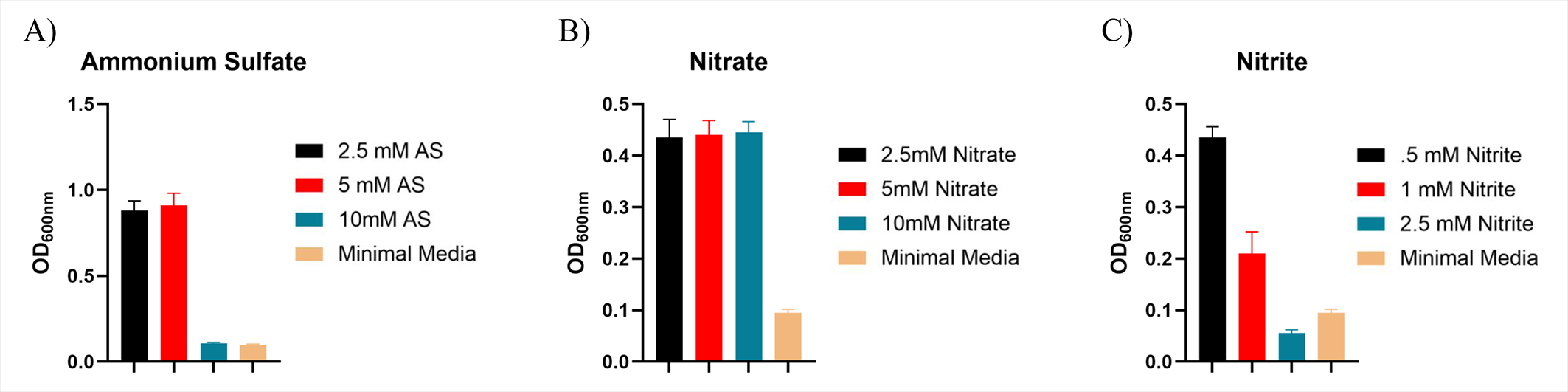

Supplement: Supplementary Figure 3 — Growth kinetics of Mab390S at different concentrations of inorganic nitrogen sources. Growth kinetics were measured by OD600nm on day 4 of growth in minimal media supplemented with (A) 2.5 mM, 5 mM, and 10 mM ammonium sulfate (B) 0.5 mM, 1 mM, 2.5 mM sodium nitrite (C) 2.5 mM, 5 mM, and 10 mM sodium nitrate. [file Image_3.tif]

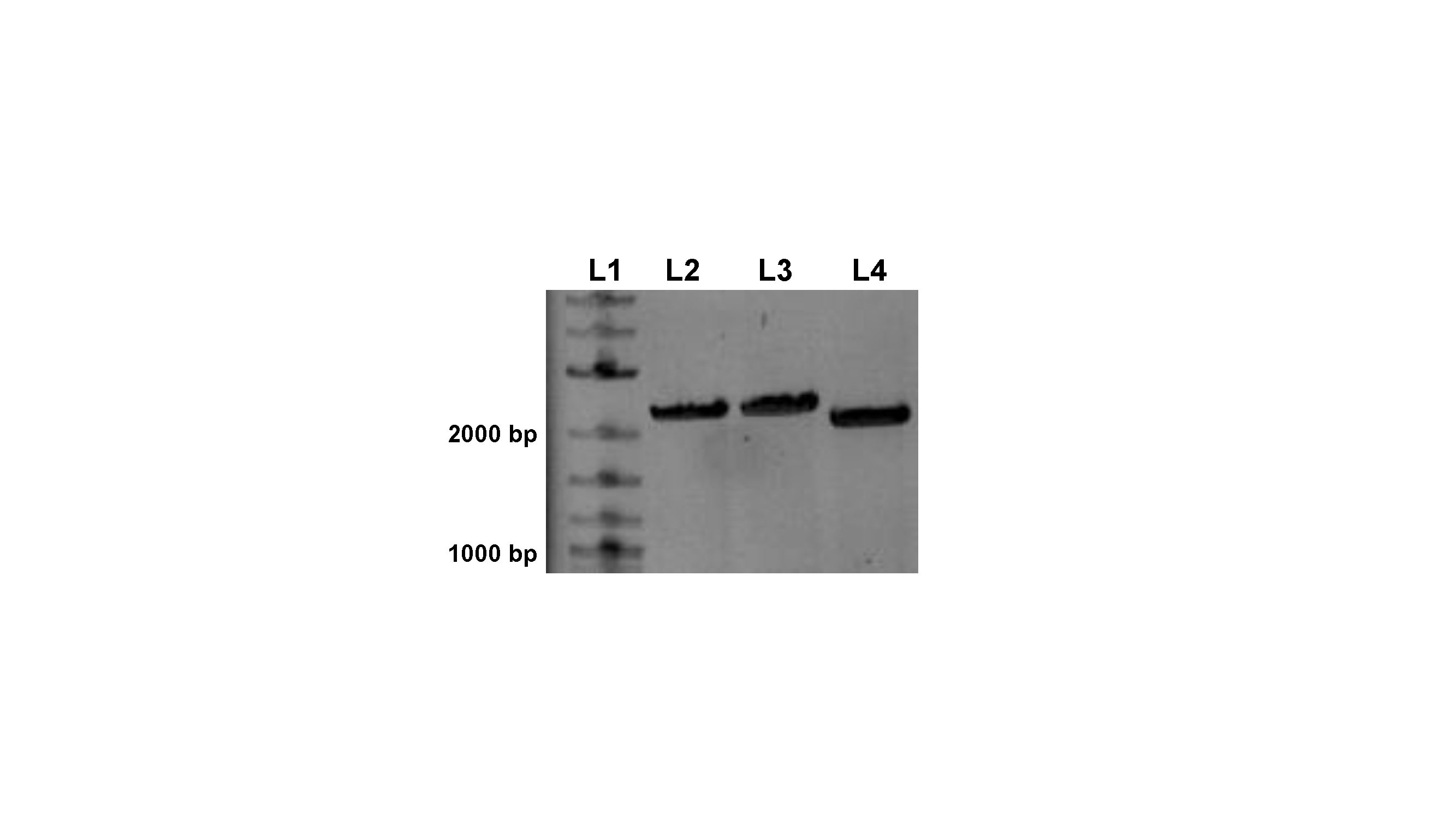

Supplement: Supplementary Figure 4 — PCR confirmation of nnaR mutant. PCR was used to screen for nnaR mutants via amplification of NnaR or Apramycin resistance cassette plus upstream and downstream flanking region. L1) DNA ladder; L2) WT Mab, 2287 bp amplicon; L3) negative clone; L4) ΔnnaR mutant, 2071 bp amplicon. [file Image_4.tiff]

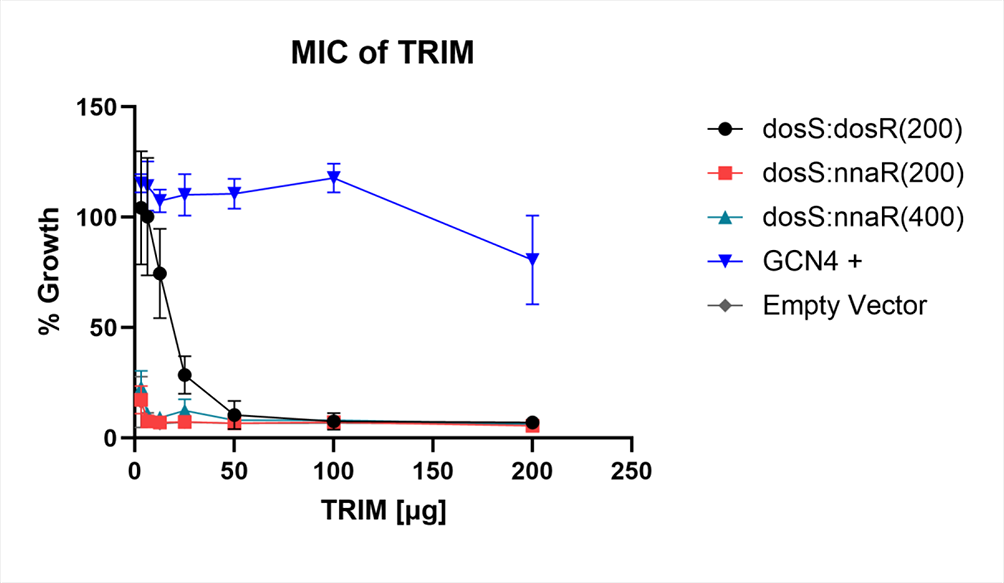

Supplement: Supplementary Figure 5 — MPFC assay displays lack of interaction between NnaR and DosS. Minimum inhibitory concentration of trimethoprim (TRIM) was analyzed to measure interaction of DHFR1,2 and DHFR3 indicative of PPI. Black circle) pUAB100-dosS-(DHFR1,2 on C-term):pUAB200-dosR-(DHFR3 on C-term) Red square) pUAB100-dosS-(DHFR1,2 on C-term):pUAB200-nnaR-(DHFR3 on C-term) Turquoise triangle) pUAB100-dosS-(DHFR1,2 on C-term):pUAB400-nnaR-(DHFR3 on N-term) Blue triangle) pUAB300-GCN4-(DHFR1,2 on C-term):pUAB400-GCN4-(DHFR3 on N-term) Gray Diamond) empty vector. [file Image_5.tif]
